# Fried Food Consumption and Cardiovascular Health: A Review of Current Evidence

**DOI:** 10.3390/nu7105404

**Published:** 2015-10-06

**Authors:** Taraka V. Gadiraju, Yash Patel, J. Michael Gaziano, Luc Djoussé

**Affiliations:** 1 The Division of Aging, Department of Medicine, Brigham and Women’s Hospital, Harvard Medical School, 1620 Tremont St, 3rd floor, Boston, MA 02120, USA; dryashpatel21@gmail.com (Y.P.); jmgaziano@partners.org (J.M.G.); ldjousse@rics.bwh.harvard.edu (L.D.); 2 Boston Veterans Affairs Healthcare System, Boston, MA 02130, USA

**Keywords:** diet, fried food, cardiovascular disease, hypertension, diabetes mellitus, obesity and risk factor

## Abstract

Fried food consumption and its effects on cardiovascular disease are still subjects of debate. The objective of this review was to summarize current evidence on the association between fried food consumption and cardiovascular disease, diabetes, hypertension and obesity and to recommend directions for future research. We used PubMed, Google Scholar and Medline searches to retrieve pertinent publications. Most available data were based on questionnaires as a tool to capture fried food intakes, and study design was limited to case-control and cohort studies. While few studies have reported a positive association between frequencies of fried food intake and risk of coronary artery disease, heart failure, diabetes or hypertension, other investigators have failed to confirm such an association. There is strong evidence suggesting a higher risk of developing chronic disease when fried foods are consumed more frequently (*i.e.*, four or more times per week). Major gaps in the current literature include a lack of detailed information on the type of oils used for frying foods, stratification of the different types of fried food, frying procedure (deep and pan frying), temperature and duration of frying, how often oils were reused and a lack of consideration of overall dietary patterns. Besides addressing these gaps, future research should also develop tools to better define fried food consumption at home *versus* away from home and to assess their effects on chronic diseases. In summary, the current review provides enough evidence to suggest adverse health effects with higher frequency of fried food consumption. While awaiting confirmation from future studies, it may be advisable to the public to consume fried foods in moderation while emphasizing an overall healthy diet.

## 1. Introduction

Chronic non-communicable diseases (NCDs), like coronary heart disease (CHD), stroke, hypertension, diabetes mellitus and obesity, are major causes of mortality and morbidity worldwide [1]. NCDs are no longer rich country problems and are a burden in both developed and developing countries [1]. The World Health Organization (WHO) projected that chronic diseases will account for almost three-quarters of all deaths worldwide by the year 2020 [2].

Diet and nutrition play a key role in the promotion and maintenance of good health, as they are important modifiable risk factors for chronic diseases. One of the leading risk factors for death and disability in the United States is suboptimal diet quality, which in 2010 was associated with 678,000 annual deaths of all causes [3]. Major contributors were insufficient intakes of fruits, nuts/seeds, whole grains, vegetables and seafood, as well as excess intakes of sodium [3]. Evidence suggests that excessive consumption of energy-dense foods high in fat, particularly saturated fat, and in refined carbohydrates can lead to weight gain, obesity and pose an increased risk for NCDs. The association between dietary fats and chronic diseases has been extensively studied with evidence indicating that dietary fats play an important role in the development of cardiovascular diseases. Hence, the focus of dietary guidelines has shifted in the last few decades from recommendations of reduced total fat intake to a greater emphasis on the type of fats consumed and the fatty acid profile of the diet. However, the evidence behind the influence of fried food consumption on long-term cardio metabolic health is unclear. This paper reviews the existing evidence on the relation of fried food consumption with major NCDs, including coronary artery disease, diabetes, hypertension and obesity.

## 2. Fried Foods and Oils

Over the last few decades, rapid economic development, mechanization and market globalization across the world have led to enormous changes in diets and lifestyles. The need for transport and a long shelf life led to the need for the modification of the natural forms of foods. Frying is a very old method of cooking and was used in the Mediterranean countries many centuries ago [4]. Frying is now famous all across the world and is a common method to produce processed food with increased durability. Moreover, frying may make food tastier with the deep-fried flavor being attractive to some consumers. The choice of frying oil is dependent on a number of factors, including cost, stability and the resistance of oil and fried food products to oxidation. The impact of the oil used and changes that occur with the industrial processes have been well reviewed in the past few years [4,5,6]. At high temperatures (150–200 °C), highly unsaturated vegetable oils have a short frying life and shelf life of the food product due to their susceptibility to oxidation. Oils rich in saturated fatty acids (SFAs) and partially hydrogenated oils have improved stability profiles for prolonged frying. Palm oil is a good frying oil, but contains 45%–50% SFA. Partially hydrogenated sunflower, rapeseed and soybean oils contain *<*20% SFA, but have high trans-fatty acids (TFA), up to 20%. The emergence of high stability (Active Oxygen Method Stability Index, AOM 35–40 h) oils, such as “non-genetically modified organism/high oleic acid” rapeseed and sunflower oils offer a viable and healthier alternative. The choice of oils also varies with the location where fried food is consumed. Most people in Spain use olive oil for frying at home [7,8]. Olive oil is highly resistant to oxidation [9,10]. In contrast, the most common oil used for frying away from home, especially in national chain fast food restaurants, is corn oil [11]. The oil absorption of the inherently low-fat and low-calorie foods makes them energy dense after frying in oil [4,5].

## 3. Dietary Fats and Chronic Diseases

Heating causes oxidative and thermal degradation, resulting in the formation of oxidized and polymerized compounds with higher polarity and, thus, modifying the nutritional properties of dietary fat and fried food. WHO recommends limiting the consumption of saturated and trans-fats (hydrogenated fats), sugars and salt in the diet, which are often found in snacks, processed foods and drinks [1]. Healthy diets should provide very low intake of TFAs (*i.e.*, <1%), adequate intake of polyunsaturated fatty acids (PUFAs) (*i.e.*, 6%–10% of daily energy intake), with an optimal balance between intake of *n*-6 PUFAs (5%–8% of daily energy intake) and *n*-3 PUFAs (1%–2% of daily energy intake) [1]. The quality of fried foods and the fatty acid composition of the final fried product largely depend on multiple factors, including actual composition of the food being fried, the type of oil used, frying conditions (temperature, duration) [4,6], *etc.*

### 3.1. Fried Food and Cardiovascular Disease (CVD)

CVD is the leading global cause of death and is projected to be associated with more than 23.6 million deaths by 2030 [3]. The low rates of CHD in various Mediterranean countries who consume a diet rich in fat (predominantly from olive oil) was reported as early as in the 1970s [7,12], but consumption of fried foods may be associated with increased risk of cardiovascular risk factors [13,14,15]. However, the evidence behind the association between consumption of fried foods and the risk of coronary artery disease (CAD) is limited and conflicting.

Although frying is generally considered an unhealthy way of preparing food [16], Guallar-Castillón and colleagues reported that consumption of fried foods was not associated with the risk of CHD after 12 years of follow-up in a prospective study of 40,757 adults aged 29–69 years free of CHD [17]. A total of 606 CHD events (rate of CHD: 1.5%) were recorded on follow-up, and no association was found between consumption of total fried food and CHD. Compared to being in the lowest quartile of fried food consumption, the multivariable-adjusted hazards ratio of CHD was 1.15 (95% CI: 0.91–1.45) in the second, 1.07 (95% CI: 0.83–1.38) in the third and 1.08 (95% CI: 0.82–1.43) in the fourth quartile of fried food consumption (*p* for trend: 0.74) [17]. No association was found between intake of fried fish (hazards ratio (HR), 1.13; 95% CI: 0.89–1.44), fried meat (HR, 1.09; 95% CI: 0.82–1.43), fried potatoes (HR, 0.90; 95% CI: 0.70–1.15) and fried eggs (HR, 0.87; 95% CI: 0.68–1.13) and CHD risk. These null findings are contrary to other studies reporting positive associations [18,19]. It is possible that Mediterranean diet patterns that include fruits and vegetables, a low intake of meat, whole grains, *etc.* [20], and/or the type of oils used for frying in Spain (mainly olive oil and sunflower oil) may partially explain the difference observed across studies. The use of extra virgin olive oil was shown to reduce lipid oxidation intensity during frying of some foods [9,10]. A case-control study from Costa Rica, including 485 survivors of a first acute myocardial infarction and 508 controls, found that an increase in the frequency of consumption of fried foods from 4.57 to 9.75 servings/day) was not associated with nonfatal acute myocardial infarction (*p* < 0.05) [21]. Palm oil, partially hydrogenated soybean oil and corn oil were the major oils used for frying in that population.

In a case-control study from India, including 165 patients with coronary heart disease and 199 matched controls, patients with coronary heart disease when compared to controls reported a greater intake of both shallow fried food (24.0 ± 60.4 *versus* 2.7 ± 17.2 g/day; *p* < 0.01) and deep-fried food (15.2 ± 25.0 *versus* 1.0 ± 5.1 g/day; *p* < 0.01) [22]. Data from a case-control study in China [23] showed that the frequency of fried food intake was significantly higher in patients with acute myocardial infarction than in controls (1.3 ± 2.1 *versus* 1.1 ± 1.8 week intake; *p* = 0.025) [23]. Data from the Nurses’ Health Study and the Health Professional Follow up Study showed that frequent fried food consumption was significantly associated with a higher risk of CAD [18]. The multivariable-adjusted relative risks (RRs, with 95% CIs) of CAD for individuals who consumed fried foods <1, 1–3, 4–6 or ≥7 times/week were 1.00 (reference), 1.06 (0.98–1.15), 1.23 (1.14–1.33) and 1.21 (1.06–1.39), respectively. The INTERHEART study (with 5761 nonfatal myocardial infarction cases and 10,646 controls from 52 countries free from angina, diabetes, hypertension or hypercholesterolemia) also observed a positive association between fried food intake and acute myocardial infarction with a multivariable-adjusted odds ratio of 1.13 (95% CI; 1.02–1.25) for the highest compared to the lowest quartile of fried food intake (*p* for trend <0.001) [19]. Results from the Cardiovascular Health Study showed that fried fish consumption is associated with trends toward higher risk of death due to ischemic heart disease [24].

Inconsistency across the above results might be partly attributed to the type and composition of the foods, the type of oil used, the frying technique (deep or pan frying) and the extent of oil degradation. More research is necessary to measure the association between fried food and CAD with improved dietary assessment focusing on the details about the type of food/oil used, frying time, temperature and the type of frying used.

### 3.2. Fried Food and Heart Failure

Fried fish has also been associated with reduced ejection fraction, lower cardiac output and higher systemic vascular resistance in older adults [25]. Belin *et al*. found that fried fish consumption (>1 serving per week at baseline) was associated with a 48% higher risk of heart failure (HF) (HR, 1.48 (95% CI: 1.19–1.84)) [26]. Djoussé *et al.* showed a positive and graded association between fried food consumption and incidence of HF in a prospective cohort study; compared to subjects who reported fried food consumption of <1 per week, the adjusted hazards ratios (95% CI) for HF were 1.24 (1.04–1.48), 1.28 (1.00–1.63) and 2.03 (1.37–3.02) for fried food intake of 1–3/week, 4–6/week and 7+/week, respectively (*p* for linear trend: 0.0002) [27]. Although existing evidence suggests a higher risk of HF in people with frequent fried food consumption, underlying biologic mechanisms remain to be elucidated.

### 3.3. Fried Food and Hypertension

Changes in lifestyle and changes in unhealthy cooking habits from frying to boiling have been found to be beneficial for controlling blood pressure and obesity in the general population [28]. There is limited and inconsistent epidemiological evidence directly relating fried food consumption and hypertension. A cross-sectional study from Spain reported that consumption of fried foods was associated with higher prevalence of hypertension [13]. The SUN (Seguimiento Universidad de Navarra) Mediterranean cohort study reported that frequent consumption of fried foods at baseline was associated with a higher risk of hypertension (adjusted hazards ratios = 1.18 (95% CI: 1.03–1.36) and 1.21 (95% CI: 1.04–1.41) for those consuming fried foods 2–4 and >4 times/week, respectively, compared to those consuming fried foods <2 times/week (*p* for trend = 0.009)) [29]. The process of oxidation during frying food increases the amount of trans-fatty acids in food and is positively associated with the risk of hypertension [30]. Wang *et al.* reported a positive association between dietary intake of trans-fatty acids and the risk of hypertension (adjusted RR in the highest quintile: 1.08; 95% CI: 1.01–1.15) [30]. Additional studies are necessary to further clarify the association between fried food consumption and hypertension along with underlying biological mechanisms.

### 3.4. Fried Food and Type 2 Diabetes (T2D)

Intake of potatoes, red meat and other processed meats has been positively associated with the risk of T2D [31,32,33,34,35]. Fried foods from restaurants and fast food consumption were positively associated with T2D [36,37,38]. Data from the Nurses' Health Study/Health Professionals Follow-Up Study revealed a strong association between the frequency of fried food consumption and the risk of T2D with adjusted RRs (95% CIs) for individuals who consumed fried foods <1, 1–3, 4–6 or ≥7 times/week of 1.00 (reference), 1.15 (0.97–1.35), 1.39 (1.30–1.49) and 1.55 (1.32–1.83), respectively [18]. The frequency of fried food consumption was also associated with the incidence of gestational diabetes (adjusted RR = 2.18 (95% CI: 1.53–3.09) comparing fried food intake of 7+ to that of <1 times per week)) [39]. However, a study from Italy demonstrated that in obese (not in lean), insulin-resistant women, consumption of foods fried in extra-virgin olive oil significantly reduced both insulin and *C*-peptide responses after a meal [40]. Taken together, there seems to be strong evidence for a positive association between fried food consumption and risk of T2D.

### 3.5. Fried Food and Obesity

Several studies reported a positive association between fried food intake and being overweight [41], waist circumference [42] or weight gain among pregnant women [43]. Frequent fried food consumption has also been reported to be associated with a higher risk of incident overweight/obesity in two large prospective studies. The European Prospective study showed a positive association of fried food consumption with central and general obesity (adjusted odds ratios for general obesity in the highest *versus* the lowest quintile of fried food intake: 1.26 (95% CI: 1.09–1.45, *p* for trend <0.001) in men and 1.25 (95% CI: 1.11–1.41, *p* for trend <0.001) in women) [44]. The SUN study also revealed similar results with an adjusted odds ratio of 1.37 (95% CI: 1.08–1.73) for developing overweight/obesity with the consumption of fried foods >4 times/week when compared to <2 times/week (*p* for trend = 0.02) [7]. More research is needed to quantify the impact of the type of oils used for frying and the frying conditions on the development of obesity.

## 4. Conclusions and Future Direction

The current review suggests that more frequent consumption of fried foods (*i.e.*, four or more times per week) is associated with a higher risk of developing T2D, HF, obesity and hypertension. Mixed results have been reported on the association of fried foods with CAD. Major gaps in the current literature on the effects of fried foods include a lack of detailed information on the type of oils used for frying foods, differentiation between the different types of fried foods, frying procedure (deep and pan frying), temperature and duration of frying, how often oils were reused and a lack of consideration of overall dietary patterns. The effects of fried food consumption at home *versus* away from home need further evaluation. Future research should focus on these gaps and help develop better assessment tools for fried food consumption and their effects on chronic diseases.

In summary, there is strong evidence suggesting an association of fried food consumption with a higher risk of developing chronic disease in adults. The strength of current evidence makes it reasonable to recommend complete avoidance of fried foods or at most infrequent to moderate fried food consumption within the context of an overall healthy dietary pattern (rich in fruits, vegetables, whole grains, low sodium, low red/processed meat, *etc.*).
